# A Successful Crayfish Invader Is Capable of Facultative Parthenogenesis: A Novel Reproductive Mode in Decapod Crustaceans

**DOI:** 10.1371/journal.pone.0020281

**Published:** 2011-05-31

**Authors:** Miloš Buřič, Martin Hulák, Antonín Kouba, Adam Petrusek, Pavel Kozák

**Affiliations:** 1 Faculty of Fisheries and Protection of Waters, South Bohemian Research Center of Aquaculture and Biodiversity of Hydrocenoses and Research Institute of Fish Culture and Hydrobiology, University of South Bohemia in Èeské Budìjovice, Vodòany, Czech Republic; 2 Department of Ecology, Faculty of Science, Charles University in Prague, Prague, Czech Republic; University of Arkanas, United States of America

## Abstract

Biological invasions are impacting biota worldwide, and explaining why some taxa tend to become invasive is of major scientific interest. North American crayfish species, particularly of the family Cambaridae, are prominent invaders in freshwaters, defying the “tens rule” which states that only a minority of species introduced to new regions become established, and only a minority of those become invasive and pests. So far, success of cambarid invaders has largely been attributed to rapid maturation, high reproductive output, aggressiveness, and tolerance to pollution. We provide experimental evidence that females of one cambarid species particularly widespread in Europe, the spiny-cheek crayfish *Orconectes limosus*, are capable of facultative parthenogenesis. Such reproductive mode has never before been recognized in decapods, the most diverse crustacean order. As shown by analysis of seven microsatellite loci, crayfish females kept physically separated from males produced genetically homogeneous offspring identical with maternal individuals; this suggests they reproduced by apomixis, unlike those females which mated with males and had a diverse offspring. Further research is needed to clarify what environmental conditions are necessary for a switch to parthenogenesis in *O. limosus*, and what role it plays in natural crayfish populations. However, if such reproductive plasticity is present in other cambarid crayfish species, it may contribute to the overwhelming invasive success of this group.

## Introduction

**Figure 1:**
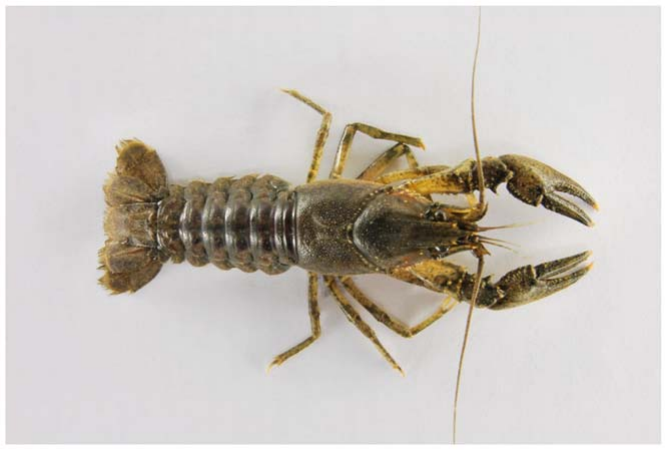
Mature female of the spiny-cheek crayfish *Orconectes limosus* from the Černovický brook (Czech Republic), a source population for our experiments. There was no difference in phenotype of females in the different experimental groups.

Crayfish are ecologically important benthic macroinvertebrates, and often act as keystone species in both standing and running waters [1]. Since they are also economically important, many crayfish species have been introduced to regions outside of their original distributions, both within and between continents. The introduction of North American crayfish to Europe has been particularly successful, but has also had serious conservational consequences, including the decimation of local crayfish populations by the crayfish plague pathogen introduced with them [2]. The first of those species, the spiny-cheek crayfish *Orconectes limosus* (Figure 1), became successfully established from a batch of 90 individuals released in 1890 to a fishpond in Pomerania (presently western Poland), and has since colonized at least 20 European countries [2], [3]. Other American crayfish have since been introduced to Europe, resulting in at least eight to nine species established at present [2], [4].

Recently, the obligate asexual marbled crayfish, later shown to be a parthenogenetic form of *Procambarus fallax* [5], was discovered in the aquarium trade [6]. Since then, it has been released to open waters and become established at several localities [7], [8]; however, asexually reproducing crayfish are not known from the original distribution of the species. Marbled crayfish, so far the only known parthenogenetic decapod crustacean, which completely lacks males, is considered to be a serious future threat to aquatic biodiversity in regions where it becomes established [7], [9], although its success in European waters seems to be constrained by climate [10]. No other crayfish (or any other decapod crustacean) proven to be capable of asexual reproduction is known from the wild.

All except one of the American crayfish invaders belong to the family Cambaridae, and their life histories are typical for r-strategists. Unlike native European astacid crayfish, many cambarids living in temperate climates, including *O. limosus*, mate twice a year, in autumn and in spring, although they lay eggs only in spring [11]. The adaptive value of this feature of their life cycle is unknown. In order to explore the impact of different mating patterns and the availability of mates to the reproductive success of *O. limosus*, and to evaluate the contribution of different males to clutch paternity, we set up laboratory experiments with individuals collected from one of the invaded localities in the Czech Republic. For this purpose, we distributed the animals into three experimental groups. Two groups consisted of thirty females and fifteen males, which were allowed visual, chemical, and tactile contact, including mating behaviour. In the third, “control” group, fifteen males were separated in a mesh cage from 30 females. Thus, the two sexes were denied the physical contact but chemical communication was allowed. Females in this group were physically isolated from males for ten months; despite that, vast majority of them successfully spawned and carried clutches of apparently viable offspring.

In this study, we analyse the genetic structure of mothers and offspring in the experiment, to evaluate possible scenarios explaining the “virgin birth” in *O. limosus*. We provide evidence that the species is capable of facultative parthenogenesis, a reproductive mode not reported before in decapod crustaceans.

## Results

Spiny-cheek crayfish females in all experimental groups, including those considered a “control” that were not expected to reproduce, laid eggs. Specifically, 28 out of thirty females in the “control” group spawned (one female died during the experiment, and only one did not lay eggs). Two of these females later lost the eggs, but the remaining 26 individuals reared viable offspring that developed normally until the 3^rd^ developmental stage when the juveniles become independent. The proportion or successfully reproducing females was fully comparable with the two “mated” groups, i.e., treatments where comparably-sized females were allowed to mate with males. In these, 27 and 25 females spawned, and subsequently 25 and 20 individuals reared viable offspring.

The number of juveniles per female in the 2^nd^ developmental stage (just before leaving the mother) was relatively high in the “control”, i.e., non-mated group (mean ± SE: 96±9.2 individuals); however, there were over 40% more juveniles successfully reared by females in the other two groups (139±8.0, and 137±8.3 juveniles, respectively). This difference between control and mating groups was highly significant (ANOVA, F = 6.88, *P* = 0.002); the two mated groups did not differ significantly between each other (Tukey post-hoc test; *P* = 0.99).

Subsequent analysis of seven variable microsatellite loci [12] for 23 females and 10 juveniles from each of their broods showed that all analysed offspring produced in the absence of males had multilocus genotypes identical to their mothers, which were often heterozygous at several—up to all seven—loci (their multilocus genotypes are given in Table S1). Females mated to males produced genetically diverse offspring; none of the analysed juveniles had a multilocus genotype identical to its mother, and they often carried alleles not observed in the maternal individual, i.e., of paternal origin (Table S2).

## Discussion

The observation that females prevented from physical contact with males for ten months successfully spawned and reared viable offspring was intriguing. Such a phenomenon could be explained by several different scenarios, most of which assume that the eggs produced by females were somehow fertilized despite physical isolation from males. At first glance, the most feasible explanation is long-term storage of sperm in the *annulus ventralis* of females, as documented in another cambarid species [13]. Although the females were kept in isolation from males for ten months beginning in August, so mating would have had to occur even earlier in the previous season, a sperm retention seemed still more likely than other possible explanations. The fact that males and females, though physically separated by a mesh and a water column, shared the same container, allows for speculation about some mechanism of sperm transfer, although such external fertilization is not known for crayfish. Alternatively, sexual reproduction might have occurred if some individuals exhibiting a female phenotype had functional male gonads. Although intersex and gynandromorph individuals have been recorded in various crayfish taxa [14], [15] and hermaphroditism seems to be common in some parastacid crayfish from the Southern Hemisphere [16], [17], none of the females selected for our experiment had any aberration in their first pleopods or other sex-related phenotypic characters. All above-mentioned scenarios have in common the fact that they assume sexual reproduction; the produced offspring would therefore be genetically diverse, and at least some juveniles would carry paternal alleles not found in their mother.

Finally, females that could not mate with males could have resorted to parthenogenetic reproduction. This scenario predicts that the offspring carry only maternal alleles; depending on the type of parthenogenesis, all juveniles might be identical to their mothers (in the case of apomixis [18]) or the maternal alleles might be recombined in the juveniles (in the case of automixis, in which meiosis is not suppressed [19], [20]). Automixis should also result in loss of heterozygosity of the parthenogenetic offspring [20], [21].

Genotyping of seven microsatellite loci clearly pointed to apomictic parthenogenesis as the most likely process observed in the “control” *O. limosus* females in our experiment. Likelihoods that genetically identical offspring are produced by sexual reproduction in several females which are heterozygous at multiple loci are extremely low (≪10^−5^); long-term sperm storage as well as other scenarios assuming sexual reproduction can therefore be ruled out. In contrast, mated females produced genetically diverse offspring as expected in sexually reproducing specimens. These results strongly suggest that *O. limosus* females under certain conditions are capable of facultative apomixis.

Facultative parthenogenesis is not a particularly rare phenomenon in animals. Clonal and sexual reproduction regularly alternates in some invertebrate groups (e.g., rotifers, cladoceran crustaceans, and aphids [22]), while in many other taxa it is an alternative strategy. In captivity, it has been documented for various vertebrates [23], even in such charismatic species such as the hammerhead shark [24] and the Komodo dragon [20]. Nevertheless, this mode of reproduction has, to our knowledge, never been observed in decapod crustaceans, the most diverse and economically most important crustacean order.

Apart from the marbled crayfish obligately reproducing by apomictic parthenogenesis [25], the only other decapod species for which a potential for asexual reproduction has been suggested (based on the observation of various individuals with identical five-microsatellite multilocus genotypes) is the red swamp crayfish *Procambarus clarkii* [26]. In the latter case, the observed genotype identities of some wild-caught crayfish could also be explained by other processes (in particular, chance sampling of sibling individuals from the same brood). However, Yue *et al.* [26] noted that all presumed natural clones were female individuals, which might support the hypothesis that parthenogenetic reproduction may occur under some circumstances in this cambarid crayfish. A major question remains why facultative asexuality has not previously been reported for the spiny-cheek crayfish, a species that has been present in Europe for more than a century and has been the subject of numerous studies.

Most likely, parthenogenetic reproduction may be triggered only under special circumstances, when males are unavailable for mating. Females mated to males in our experiment had significantly larger clutch sizes than those reproducing parthenogenetically; this suggests some costs to asexuality. However, a vast majority of females in the “control” group switched to parthenogenesis successfully, apparently responding to some environmental cues. The role of different factors remains to be explored by further experimental work; however, we suggest several that may play a role in switching between alternative reproductive strategies. First, if infochemicals released from males during the mating season indicate the presence of the opposite sex but mating is not possible due to physical isolation, females not finding mates during the suitable period (despite sensing their presence) may resort to parthenogenesis rather than skipping one reproductive season entirely. In addition to this factor, intrasexual chemical communication between numerous females in a confined space, indicating “crowding” of this sex, could contribute to the switch to parthenogenesis. Last but not least, adequate abiotic conditions (such as light and temperature regimes corresponding to the mating season) may also be necessary for triggering parthenogenesis in both natural and artificial conditions. It is possible that a combination of several different factors is required for a successful switch to asexual reproduction, which limits the likelihood that the phenomenon occurs in laboratory cultures. Nevertheless, we had already previously observed the spawning of *O. limosus* females isolated from males under similar conditions (ambient temperature and photoperiod, sexes physically separated but in tanks connected by a flow-through system). At that time, however, we did not further examine these females or their broods.

The fact that this phenomenon was initially overlooked even by our group focused on crayfish biology brings up another likely explanation for the lack of data on parthenogenetic reproduction in cambarid crayfish: earlier casual observers of facultative parthenogenesis may have just dismissed the observation as an experimental artefact (in particular, a previously undetected mating). It has generally been stated that crayfish do not reproduce asexually, so any case when apparently unmated females released eggs would likely have been attributed to sperm storage in the *annulus ventralis*, which had been documented earlier [13]. Similarly, indirect evidence of the potential contribution of asexual reproduction to the genetic structure of natural populations, such as Hardy-Weinberg disequilibrium, may have other more feasible explanations (the Wahlund effect, the presence of null alleles, assortative mating, etc.).

Our experiment, complemented by the genetic analysis of variable nuclear markers, proves that at least one species of cambarid crayfish is capable of facultative parthenogenesis. The actual prevalence of this phenomenon under natural conditions is not known. However, it is possible that this reproductive mode is more widespread among cambarids, and contributes to the success of this group when colonizing new habitats and territories. Asexual reproduction might also have contributed to observations of putative *P. clarkii* clones [26], and possibly to significant heterozygote deficiencies observed in Chinese populations of *P. clarkii* [27] as well as in some recently established invasive populations of *O. limosus* in the Czech Republic [3], so far explained by founder effects or assortative mating. However, despite the apparent ability of *O. limosus* females to reproduce clonally, most studied populations in Europe have been in Hardy-Weinberg equilibrium [3], [12], and asexually reproducing individuals have so far escaped attention. Apparently, sexual reproduction in this species strongly dominates, or is the only reproduction mode in established populations where males are present. It remains to be tested whether females of this species are able to reproduce entirely without males; so far, there has been no report of female-only populations of any cambarid with the exception of established marbled crayfish populations, for which asexuality is the sole reproduction mode [7], [8].

The fact that unmated spiny-cheek crayfish females produced a significantly lower number of eggs is notable. It is possible that although less numerous, clonally-produced eggs are larger than those from mated females; such adjustments in female primary reproductive investment have been reported in crayfish [28]. Alternatively, clonal offspring could be less fit, e.g., due to elevated egg mortality. Facultative parthenogenetic reproduction might have also promoted the evolution of the prolonged or double mating season in cambarids. This life history trait could increase the likelihood of females finding mates before resorting to asexual reproduction as an alternative strategy that leads to lower offspring numbers; double mating may also serve as means of increasing the genetic diversity of the offspring by multiple paternity.

To conclude, we can state that *O. limosus* females are able, at least under certain circumstances, to reproduce asexually by apomictic parthenogenesis, similarly as in the parthenogenetic form of *Procambarus fallax*, the marbled crayfish. However, the latter reproduces obligately asexually, while the observed asexuality in *O. limosus* is to our knowledge the first evidence for facultative parthenogenesis in decapods. We assume that at least some other cambarid crayfish may also be capable of switching to this reproductive mode. The capability of asexual reproduction may increase the chances of withstanding unfavourable conditions when sexual reproduction is not possible, or colonising new habitats from a small initial number of specimens.

While it remains unclear whether a single or several females of facultative parthenogenetic crayfish can establish a viable population without males, the reproductive plasticity of the spiny-cheek crayfish (and possibly other cambarids) apparently makes them less prone to various eradication techniques. For example, the sterile male release technique, recently suggested to be efficient for *Procambarus clarkii* [29], may be ineffective for lower population densities if females switch to clonal reproduction instead of seeking mates, or after mating with a sterile mate. Our findings confirm that cambarid crayfish, a serious threat to not only European freshwater ecosystems, are extremely well adapted to invasions.

## Materials and Methods

### Experimental setup

*Orconectes limosus* specimens were captured in the Černovický brook (South Bohemia, Czech Republic; 49°15′51″N, 14°43′02″E) in August 2007. They were acclimated (sexes separated) to laboratory conditions for one month and placed in experimental tanks in September 2007. Ninety mature females and 45 mature males, randomly selected from these captured and acclimated animals, were used in the experiment. All these specimens were in the sexually active form I (in accordance with glair gland development in females, and the presence of form I copulatory stylets in males [30], [31]) at the beginning of the experiment. The animals were divided into three experimental groups. Two groups consisted of thirty females and fifteen males that were allowed to move freely in their tanks. Visual, chemical, and tactile contact, including mating behaviour, was thus possible. In the third, “control” tank, fifteen males were separated from the 30 females by being placed in a cage made of fine, ∼3 mm plastic mesh, suspended under the water surface, about 30 cm above the container bottom. This prevented physical contact between males and females but allowed chemical communication between the sexes. Altogether, females in this group were physically isolated from males for a period of 10 months (from August 2007 to the end of the experiment in June 2008). Female sizes (cephalothorax length) did not differ significantly among experimental groups (average ± SD: 31.8±3.40 mm, 32.4±2.71 mm, 31.2±2.87 mm; ANOVA, F = 0.98; *P* = 0.38).

The experimental tanks were circular (0.6 m diameter, depth 0.6 m, volume 0.18 m^3^), supplied with ∼3 shelters per crayfish. Males placed in the cage suspended in the “control” tank had one shelter per individual. Photoperiod and water temperature were ambient, provided by natural daylight and a flow-through water supply. Tanks were cleaned regularly, and dissolved oxygen was measured twice a day using an oximeter (Oxi 315i, WTW GmbH, Weilheim, Germany). Water temperature was measured every three hours using dataloggers (RT-F53, Qi Analytical, Prague, Czech Republic). The pH was measured daily (pH 315i, WTW GmbH, Weilheim, Germany). Crayfish were fed to satiation on fish pellets, frozen chironomid larvae, and carrots two to five times per week (depending on the season and the amount of food left uneaten).

After spawning was finished for all groups in spring, males were removed and females were kept separately until the end of rearing, i.e., when juveniles reached the 2^nd^ developmental stage [32]. The number of juveniles was counted after stripping from the female's pleopods. Forty juveniles were collected from each successfully reproducing female, and preserved in pure ethanol (96%). The remaining juveniles were then reared as separated groups to observe their viability (fed by live and frozen zooplankton in excess) but were discarded approximately one month later.

### *Genotyping*

Tissue samples were obtained from each analyzed female crayfish individual (all females from the control group that reared young, and ten randomly selected females from the other two groups) and their offspring (10 juveniles per female). DNA was extracted from muscle tissue using the E.Z.N.A. Tissue DNA Mini Kit (Peqlab Biotechnologie). Individuals were genotyped at seven microsatellite loci (PclG-2, PclG-26, PclG-8, 2.12, PclG-37, PclG-24, 3.1) using published thermal cycling conditions and primers [12]. Approximately 50 ng of DNA was used for a 15 µl PCR containing 5 µl of PPP master mix (Top-bio; [150 mM Tris-HCl, pH 8.8, 40 mM (NH_4_)_2_SO_4_, 0.02% Tween 20, 5 mM MgCl_2_, 400 µM of each dNTP, 0.1 U/µl Taq-Purple DNA-polymerase]), 0.3 µl of each primer (10 pmol/µl), 1 µl genomic DNA, and sterile water to a final volume of 15 µl. PCR amplifications were performed with one of the two primers for a given locus labelled with WellRed fluorescence dyes (Proligo), to enable the determination of allele sizes on a CEQ 2000 XL (Beckman Coulter) automated DNA sequencer using a 400 bp internal size standard. PCR and electrophoretic separation were repeated twice and provided unambiguous genotypes for each individual, identical over both independent genotyping runs. We successfully amplified all seven loci from 23 females from the “control” group, and therefore proceeded with genotyping of their offspring. For three females, amplification was successful for three loci only; these were therefore excluded from further analyses.

## Supporting Information

Table S1 Multilocus genotypes of 23 spiny-cheek crayfish females that apparently reproduced by apomictic parthenogenesis, and their offspring, for which amplification of all seven microsatellite loci was successful. Alleles are given as fragment sizes in base pairs. Note that female no. 10 was heterozygous at all analyzed loci. All analyzed juveniles of these females had multilocus genotypes identical to their mothers, so only the first three juvenile genotypes are shown.
(DOC)

Table S2 An example of allelic inheritance after sexual reproduction in spiny-cheek crayfish. Multilocus genotypes for 5 females, 5 males (candidate fathers) and their offspring (juveniles) are presented as the sizes (in base pairs) of alleles at seven microsatellite loci.
(DOC)
