# Supplementary material for: A Successful Crayfish Invader Is Capable of Facultative Parthenogenesis: A Novel Reproductive Mode in Decapod Crustaceans
Source: PLoS One. 2011 May 31;6(5):e20281. doi: 10.1371/journal.pone.0020281 (PMC3105005; doi:10.1371/journal.pone.0020281)
Supplement: Table S1 — Multilocus genotypes of 23 spiny-cheek crayfish females that apparently reproduced by apomictic parthenogenesis, and their offspring, for which amplification of all seven microsatellite loci was successful. Alleles are given as fragment sizes in base pairs. Note that female no. 10 was heterozygous at all analyzed loci. All analyzed juveniles of these females had multilocus genotypes identical to their mothers, so only the first three juvenile genotypes are shown. (DOC) [file pone.0020281.s001.doc]

|  | **Allele sizes (bp) for seven microsatellite loci** | | | | | | | | | | | | | |
| --- | --- | --- | --- | --- | --- | --- | --- | --- | --- | --- | --- | --- | --- | --- |
| *Locus* | *PclG-2* | | *PclG-26* | | *PclG-8* | | *2.12* | | *PclG-37* | | *PclG-24* | | *3.1* | |
| *Female 1* | 297 | 297 | 278 | 283 | 195 | 225 | 158 | 158 | 147 | 161 | 224 | 229 | 297 | 297 |
| juvenile | 297 | 297 | 278 | 283 | 195 | 225 | 158 | 158 | 147 | 161 | 224 | 229 | 297 | 297 |
| juvenile | 297 | 297 | 278 | 283 | 195 | 225 | 158 | 158 | 147 | 161 | 224 | 229 | 297 | 297 |
| juvenile | 297 | 297 | 278 | 283 | 195 | 225 | 158 | 158 | 147 | 161 | 224 | 229 | 297 | 297 |
| remaining Female 1 juveniles had the same genotype | | | | | | | | | | | | | | |
| *Female 2* | 297 | 306 | 281 | 283 | 195 | 225 | 158 | 158 | 147 | 161 | 224 | 229 | 293 | 297 |
| juvenile | 297 | 306 | 281 | 283 | 195 | 225 | 158 | 158 | 147 | 161 | 224 | 229 | 293 | 297 |
| juvenile | 297 | 306 | 281 | 283 | 195 | 225 | 158 | 158 | 147 | 161 | 224 | 229 | 293 | 297 |
| juvenile | 297 | 306 | 281 | 283 | 195 | 225 | 158 | 158 | 147 | 161 | 224 | 229 | 293 | 297 |
| remaining Female 2 juveniles had the same genotype | | | | | | | | | | | | | | |
| *Female 3* | 297 | 301 | 278 | 282 | 195 | 225 | 158 | 158 | 147 | 163 | 224 | 224 | 291 | 295 |
| juvenile | 297 | 301 | 278 | 282 | 195 | 225 | 158 | 158 | 147 | 163 | 224 | 224 | 291 | 295 |
| juvenile | 297 | 301 | 278 | 282 | 195 | 225 | 158 | 158 | 147 | 163 | 224 | 224 | 291 | 295 |
| juvenile | 297 | 301 | 278 | 282 | 195 | 225 | 158 | 158 | 147 | 163 | 224 | 224 | 291 | 295 |
| remaining Female 3 juveniles had the same genotype | | | | | | | | | | | | | | |
| *Female 4* | 297 | 301 | 278 | 282 | 195 | 225 | 158 | 158 | 147 | 147 | 208 | 208 | 297 | 301 |
| juvenile | 297 | 301 | 278 | 282 | 195 | 225 | 158 | 158 | 147 | 147 | 208 | 208 | 297 | 301 |
| juvenile | 297 | 301 | 278 | 282 | 195 | 225 | 158 | 158 | 147 | 147 | 208 | 208 | 297 | 301 |
| juvenile | 297 | 301 | 278 | 282 | 195 | 225 | 158 | 158 | 147 | 147 | 208 | 208 | 297 | 301 |
| remaining Female 4 juveniles had the same genotype | | | | | | | | | | | | | | |
| *Female 5* | 297 | 301 | 278 | 285 | 171 | 195 | 145 | 158 | 153 | 153 | 208 | 222 | 297 | 301 |
| juvenile | 297 | 301 | 278 | 285 | 171 | 195 | 145 | 158 | 153 | 153 | 208 | 222 | 297 | 301 |
| juvenile | 297 | 301 | 278 | 285 | 171 | 195 | 145 | 158 | 153 | 153 | 208 | 222 | 297 | 301 |
| juvenile | 297 | 301 | 278 | 285 | 171 | 195 | 145 | 158 | 153 | 153 | 208 | 222 | 297 | 301 |
| remaining Female 5 juveniles had the same genotype | | | | | | | | | | | | | | |
| *Female 6* | 297 | 297 | 278 | 282 | 195 | 195 | 158 | 158 | 151 | 153 | 208 | 227 | 293 | 293 |
| juvenile | 297 | 297 | 278 | 282 | 195 | 195 | 158 | 158 | 151 | 153 | 208 | 227 | 293 | 293 |
| juvenile | 297 | 297 | 278 | 282 | 195 | 195 | 158 | 158 | 151 | 153 | 208 | 227 | 293 | 293 |
| juvenile | 297 | 297 | 278 | 282 | 195 | 195 | 158 | 158 | 151 | 153 | 208 | 227 | 293 | 293 |
| remaining Female 6 juveniles had the same genotype | | | | | | | | | | | | | | |
| *Female 7* | 297 | 304 | 278 | 278 | 171 | 195 | 158 | 158 | 147 | 153 | 224 | 227 | 293 | 293 |
| juvenile | 297 | 304 | 278 | 278 | 171 | 195 | 158 | 158 | 147 | 153 | 224 | 227 | 293 | 293 |
| juvenile | 297 | 304 | 278 | 278 | 171 | 195 | 158 | 158 | 147 | 153 | 224 | 227 | 293 | 293 |
| juvenile | 297 | 304 | 278 | 278 | 171 | 195 | 158 | 158 | 147 | 153 | 224 | 227 | 293 | 293 |
| remaining Female 7 juveniles had the same genotype | | | | | | | | | | | | | | |
| *Female 8* | 297 | 301 | 278 | 278 | 195 | 195 | 158 | 158 | 147 | 147 | 208 | 227 | 297 | 301 |
| juvenile | 297 | 301 | 278 | 278 | 195 | 195 | 158 | 158 | 147 | 147 | 208 | 227 | 297 | 301 |
| juvenile | 297 | 301 | 278 | 278 | 195 | 195 | 158 | 158 | 147 | 147 | 208 | 227 | 297 | 301 |
| juvenile | 297 | 301 | 278 | 278 | 195 | 195 | 158 | 158 | 147 | 147 | 208 | 227 | 297 | 301 |
| remaining Female 8 juveniles had the same genotype | | | | | | | | | | | | | | |
| *Female 9* | 297 | 297 | 278 | 283 | 195 | 195 | 158 | 158 | 151 | 153 | 208 | 227 | 297 | 301 |
| juvenile | 297 | 297 | 278 | 283 | 195 | 195 | 158 | 158 | 151 | 153 | 208 | 227 | 297 | 301 |
| juvenile | 297 | 297 | 278 | 283 | 195 | 195 | 158 | 158 | 151 | 153 | 208 | 227 | 297 | 301 |
| juvenile | 297 | 297 | 278 | 283 | 195 | 195 | 158 | 158 | 151 | 153 | 208 | 227 | 297 | 301 |
| remaining Female 9 juveniles had the same genotype | | | | | | | | | | | | | | |
| *Female 10* | 297 | 301 | 278 | 285 | 171 | 195 | 145 | 158 | 151 | 153 | 208 | 222 | 297 | 301 |
| juvenile | 297 | 301 | 278 | 285 | 171 | 195 | 145 | 158 | 151 | 153 | 208 | 222 | 297 | 301 |
| juvenile | 297 | 301 | 278 | 285 | 171 | 195 | 145 | 158 | 151 | 153 | 208 | 222 | 297 | 301 |
| juvenile | 297 | 301 | 278 | 285 | 171 | 195 | 145 | 158 | 151 | 153 | 208 | 222 | 297 | 301 |
| remaining Female 10 juveniles had the same genotype | | | | | | | | | | | | | | |
| *Female 11* | 301 | 301 | 278 | 278 | 171 | 195 | 158 | 158 | 147 | 147 | 208 | 229 | 291 | 295 |
| juvenile | 301 | 301 | 278 | 278 | 171 | 195 | 158 | 158 | 147 | 147 | 208 | 229 | 291 | 295 |
| juvenile | 301 | 301 | 278 | 278 | 171 | 195 | 158 | 158 | 147 | 147 | 208 | 229 | 291 | 295 |
| juvenile | 301 | 301 | 278 | 278 | 171 | 195 | 158 | 158 | 147 | 147 | 208 | 229 | 291 | 295 |
| remaining Female 11 juveniles had the same genotype | | | | | | | | | | | | | | |
| *Female 12* | 297 | 297 | 278 | 285 | 195 | 225 | 158 | 158 | 147 | 147 | 208 | 229 | 291 | 295 |
| juvenile | 297 | 297 | 278 | 285 | 195 | 225 | 158 | 158 | 147 | 147 | 208 | 229 | 291 | 295 |
| juvenile | 297 | 297 | 278 | 285 | 195 | 225 | 158 | 158 | 147 | 147 | 208 | 229 | 291 | 295 |
| juvenile | 297 | 297 | 278 | 285 | 195 | 225 | 158 | 158 | 147 | 147 | 208 | 229 | 291 | 295 |
| remaining Female 12 juveniles had the same genotype | | | | | | | | | | | | | | |
| *Female 13* | 301 | 301 | 278 | 278 | 195 | 225 | 158 | 158 | 147 | 147 | 208 | 229 | 295 | 301 |
| juvenile | 301 | 301 | 278 | 278 | 195 | 225 | 158 | 158 | 147 | 147 | 208 | 229 | 295 | 301 |
| juvenile | 301 | 301 | 278 | 278 | 195 | 225 | 158 | 158 | 147 | 147 | 208 | 229 | 295 | 301 |
| juvenile | 301 | 301 | 278 | 278 | 195 | 225 | 158 | 158 | 147 | 147 | 208 | 229 | 295 | 301 |
| remaining Female 13 juveniles had the same genotype | | | | | | | | | | | | | | |
| *Female 14* | 306 | 306 | 278 | 278 | 195 | 225 | 145 | 145 | 151 | 161 | 222 | 229 | 297 | 301 |
| juvenile | 306 | 306 | 278 | 278 | 195 | 225 | 145 | 145 | 151 | 161 | 222 | 229 | 297 | 301 |
| juvenile | 306 | 306 | 278 | 278 | 195 | 225 | 145 | 145 | 151 | 161 | 222 | 229 | 297 | 301 |
| juvenile | 306 | 306 | 278 | 278 | 195 | 225 | 145 | 145 | 151 | 161 | 222 | 229 | 297 | 301 |
| remaining Female 14 juveniles had the same genotype | | | | | | | | | | | | | | |
| *Female 15* | 306 | 306 | 274 | 283 | 171 | 171 | 145 | 158 | 151 | 161 | 222 | 229 | 291 | 291 |
| juvenile | 306 | 306 | 274 | 283 | 171 | 171 | 145 | 158 | 151 | 161 | 222 | 229 | 291 | 291 |
| juvenile | 306 | 306 | 274 | 283 | 171 | 171 | 145 | 158 | 151 | 161 | 222 | 229 | 291 | 291 |
| juvenile | 306 | 306 | 274 | 283 | 171 | 171 | 145 | 158 | 151 | 161 | 222 | 229 | 291 | 291 |
| remaining Female 15 juveniles had the same genotype | | | | | | | | | | | | | | |
| *Female 16* | 292 | 294 | 274 | 274 | 171 | 171 | 145 | 158 | 151 | 161 | 222 | 229 | 297 | 297 |
| juvenile | 292 | 294 | 274 | 274 | 171 | 171 | 145 | 158 | 151 | 161 | 222 | 229 | 297 | 297 |
| juvenile | 292 | 294 | 274 | 274 | 171 | 171 | 145 | 158 | 151 | 161 | 222 | 229 | 297 | 297 |
| juvenile | 292 | 294 | 274 | 274 | 171 | 171 | 145 | 158 | 151 | 161 | 222 | 229 | 297 | 297 |
| remaining Female 16 juveniles had the same genotype | | | | | | | | | | | | | | |
| *Female 17* | 292 | 298 | 274 | 274 | 171 | 225 | 158 | 158 | 147 | 161 | 222 | 229 | 301 | 301 |
| juvenile | 292 | 298 | 274 | 274 | 171 | 225 | 158 | 158 | 147 | 161 | 222 | 229 | 301 | 301 |
| juvenile | 292 | 298 | 274 | 274 | 171 | 225 | 158 | 158 | 147 | 161 | 222 | 229 | 301 | 301 |
| juvenile | 292 | 298 | 274 | 274 | 171 | 225 | 158 | 158 | 147 | 161 | 222 | 229 | 301 | 301 |
| remaining Female 17 juveniles had the same genotype | | | | | | | | | | | | | | |
| *Female 18* | 294 | 302 | 274 | 285 | 195 | 195 | 158 | 158 | 147 | 147 | 222 | 227 | 297 | 297 |
| juvenile | 294 | 302 | 274 | 285 | 195 | 195 | 158 | 158 | 147 | 147 | 222 | 227 | 297 | 297 |
| juvenile | 294 | 302 | 274 | 285 | 195 | 195 | 158 | 158 | 147 | 147 | 222 | 227 | 297 | 297 |
| juvenile | 294 | 302 | 274 | 285 | 195 | 195 | 158 | 158 | 147 | 147 | 222 | 227 | 297 | 297 |
| remaining Female 18 juveniles had the same genotype | | | | | | | | | | | | | | |
| *Female 19* | 294 | 297 | 274 | 285 | 225 | 225 | 158 | 158 | 147 | 153 | 222 | 227 | 297 | 297 |
| juvenile | 294 | 297 | 274 | 285 | 225 | 225 | 158 | 158 | 147 | 153 | 222 | 227 | 297 | 297 |
| juvenile | 294 | 297 | 274 | 285 | 225 | 225 | 158 | 158 | 147 | 153 | 222 | 227 | 297 | 297 |
| juvenile | 294 | 297 | 274 | 285 | 225 | 225 | 158 | 158 | 147 | 153 | 222 | 227 | 297 | 297 |
| remaining Female 19 juveniles had the same genotype | | | | | | | | | | | | | | |
| *Female 20* | 294 | 306 | 274 | 285 | 171 | 225 | 145 | 145 | 161 | 161 | 222 | 229 | 297 | 301 |
| juvenile | 294 | 306 | 274 | 285 | 171 | 225 | 145 | 145 | 161 | 161 | 222 | 229 | 297 | 301 |
| juvenile | 294 | 306 | 274 | 285 | 171 | 225 | 145 | 145 | 161 | 161 | 222 | 229 | 297 | 301 |
| juvenile | 294 | 306 | 274 | 285 | 171 | 225 | 145 | 145 | 161 | 161 | 222 | 229 | 297 | 301 |
| remaining Female 20 juveniles had the same genotype | | | | | | | | | | | | | | |
| *Female 21* | 294 | 306 | 274 | 274 | 171 | 225 | 145 | 145 | 161 | 161 | 222 | 229 | 297 | 301 |
| juvenile | 294 | 306 | 274 | 274 | 171 | 225 | 145 | 145 | 161 | 161 | 222 | 229 | 297 | 301 |
| juvenile | 294 | 306 | 274 | 274 | 171 | 225 | 145 | 145 | 161 | 161 | 222 | 229 | 297 | 301 |
| juvenile | 294 | 306 | 274 | 274 | 171 | 225 | 145 | 145 | 161 | 161 | 222 | 229 | 297 | 301 |
| remaining Female 21 juveniles had the same genotype | | | | | | | | | | | | | | |
| *Female 22* | 297 | 301 | 274 | 274 | 171 | 225 | 145 | 158 | 161 | 161 | 208 | 227 | 291 | 295 |
| juvenile | 297 | 301 | 274 | 274 | 171 | 225 | 145 | 158 | 161 | 161 | 208 | 227 | 291 | 295 |
| juvenile | 297 | 301 | 274 | 274 | 171 | 225 | 145 | 158 | 161 | 161 | 208 | 227 | 291 | 295 |
| juvenile | 297 | 301 | 274 | 274 | 171 | 225 | 145 | 158 | 161 | 161 | 208 | 227 | 291 | 295 |
| remaining Female 22 juveniles had the same genotype | | | | | | | | | | | | | | |
| *Female 23* | 294 | 306 | 274 | 274 | 171 | 225 | 145 | 158 | 147 | 151 | 208 | 227 | 291 | 295 |
| juvenile | 294 | 306 | 274 | 274 | 171 | 225 | 145 | 158 | 147 | 151 | 208 | 227 | 291 | 295 |
| juvenile | 294 | 306 | 274 | 274 | 171 | 225 | 145 | 158 | 147 | 151 | 208 | 227 | 291 | 295 |
| juvenile | 294 | 306 | 274 | 274 | 171 | 225 | 145 | 158 | 147 | 151 | 208 | 227 | 291 | 295 |
| remaining Female 23 juveniles had the same genotype | | | | | | | | | | | | | | |
