# Supplementary material for: A Successful Crayfish Invader Is Capable of Facultative Parthenogenesis: A Novel Reproductive Mode in Decapod Crustaceans
Source: PLoS One. 2011 May 31;6(5):e20281. doi: 10.1371/journal.pone.0020281 (PMC3105005; doi:10.1371/journal.pone.0020281)
Supplement: Table S2 — An example of allelic inheritance after sexual reproduction in spiny-cheek crayfish. Multilocus genotypes for 5 females, 5 males (candidate fathers) and their offspring (juveniles) are presented as the sizes (in base pairs) of alleles at seven microsatellite loci. (DOC) [file pone.0020281.s002.doc]

|  | **Allele sizes (bp) for seven microsatellite loci** | | | | | | | | | | | | | |
| --- | --- | --- | --- | --- | --- | --- | --- | --- | --- | --- | --- | --- | --- | --- |
| *Locus* | *PclG-2* | | *PclG-26* | | *PclG-8* | | *2.12* | | *PclG-37* | | *PclG-24* | | *3.1* | |
| *Female A* | 297 | 297 | 278 | 283 | 195 | 225 | 158 | 158 | 147 | 161 | 227 | 227 | 291 | 295 |
| *Male 1* | 297 | 297 | 281 | 285 | 195 | 195 | 145 | 145 | 147 | 161 | 227 | 227 | 295 | 301 |
| juvenile | 297 | 297 | 281 | 283 | 195 | 195 | 145 | 158 | 147 | 161 | 227 | 227 | 291 | 301 |
| juvenile | 297 | 297 | 283 | 285 | 195 | 195 | 145 | 158 | 147 | 161 | 227 | 227 | 295 | 295 |
| juvenile | 297 | 297 | 281 | 283 | 195 | 195 | 145 | 158 | 147 | 161 | 227 | 227 | 291 | 301 |
| juvenile | 297 | 297 | 283 | 285 | 195 | 195 | 145 | 158 | 147 | 161 | 227 | 227 | 295 | 295 |
| juvenile | 297 | 297 | 281 | 283 | 195 | 195 | 145 | 158 | 147 | 161 | 227 | 227 | 295 | 295 |
| juvenile | 297 | 297 | 283 | 285 | 195 | 195 | 145 | 158 | 147 | 161 | 227 | 227 | 295 | 295 |
| juvenile | 297 | 297 | 281 | 283 | 195 | 195 | 145 | 158 | 147 | 161 | 227 | 227 | 291 | 301 |
| juvenile | 297 | 297 | 283 | 285 | 195 | 195 | 145 | 158 | 147 | 161 | 227 | 227 | 295 | 295 |
| juvenile | 297 | 297 | 281 | 283 | 195 | 195 | 145 | 158 | 147 | 161 | 227 | 227 | 291 | 301 |
| juvenile | 297 | 297 | 283 | 285 | 195 | 195 | 145 | 158 | 147 | 161 | 227 | 227 | 295 | 295 |
| *Female B* | 297 | 297 | 278 | 283 | 195 | 229 | 158 | 158 | 147 | 161 | 227 | 227 | 291 | 295 |
| *Male 2* | 294 | 301 | 274 | 274 | 225 | 225 | 145 | 145 | 144 | 161 | 227 | 227 | 295 | 295 |
| juvenile | 294 | 297 | 274 | 283 | 195 | 225 | 145 | 158 | 144 | 161 | 227 | 227 | 295 | 295 |
| juvenile | 297 | 301 | 274 | 278 | 225 | 229 | 145 | 158 | 161 | 161 | 227 | 227 | 291 | 295 |
| juvenile | 297 | 301 | 274 | 278 | 225 | 229 | 145 | 158 | 147 | 161 | 227 | 227 | 291 | 295 |
| juvenile | 297 | 301 | 274 | 278 | 225 | 229 | 145 | 158 | 147 | 161 | 227 | 227 | 291 | 295 |
| juvenile | 294 | 297 | 274 | 283 | 195 | 225 | 145 | 158 | 144 | 161 | 227 | 227 | 295 | 295 |
| juvenile | 297 | 301 | 274 | 278 | 225 | 229 | 145 | 158 | 161 | 161 | 227 | 227 | 291 | 295 |
| juvenile | 297 | 301 | 274 | 278 | 225 | 229 | 145 | 158 | 147 | 161 | 227 | 227 | 291 | 295 |
| juvenile | 297 | 301 | 274 | 278 | 225 | 229 | 145 | 158 | 147 | 161 | 227 | 227 | 291 | 295 |
| juvenile | 294 | 297 | 274 | 283 | 195 | 225 | 145 | 158 | 144 | 161 | 227 | 227 | 295 | 295 |
| juvenile | 297 | 301 | 274 | 278 | 225 | 229 | 145 | 158 | 161 | 161 | 227 | 227 | 291 | 295 |
| *Female C* | 294 | 294 | 274 | 274 | 225 | 225 | 158 | 158 | 147 | 161 | 208 | 208 | 295 | 295 |
| *Male 3* | 304 | 304 | 274 | 274 | 225 | 237 | 158 | 158 | 147 | 163 | 208 | 208 | 295 | 295 |
| juvenile | 294 | 304 | 274 | 274 | 225 | 237 | 158 | 158 | 147 | 163 | 208 | 208 | 295 | 295 |
| juvenile | 294 | 304 | 274 | 274 | 225 | 237 | 158 | 158 | 147 | 163 | 208 | 208 | 295 | 295 |
| juvenile | 294 | 304 | 274 | 274 | 225 | 237 | 158 | 158 | 147 | 163 | 208 | 208 | 295 | 295 |
| juvenile | 294 | 304 | 274 | 274 | 225 | 237 | 158 | 158 | 147 | 163 | 208 | 208 | 295 | 295 |
| juvenile | 294 | 304 | 274 | 274 | 225 | 225 | 158 | 158 | 147 | 163 | 208 | 208 | 295 | 295 |
| juvenile | 294 | 304 | 274 | 274 | 225 | 225 | 158 | 158 | 147 | 163 | 208 | 208 | 295 | 295 |
| juvenile | 294 | 304 | 274 | 274 | 225 | 225 | 158 | 158 | 147 | 163 | 208 | 208 | 295 | 295 |
| juvenile | 294 | 304 | 274 | 274 | 225 | 237 | 158 | 158 | 147 | 163 | 208 | 208 | 295 | 295 |
| juvenile | 294 | 304 | 274 | 274 | 225 | 237 | 158 | 158 | 147 | 163 | 208 | 208 | 295 | 295 |
| juvenile | 294 | 304 | 274 | 274 | 225 | 237 | 158 | 158 | 147 | 163 | 208 | 208 | 295 | 295 |
| *Female D* | 294 | 294 | 283 | 283 | 225 | 225 | 145 | 145 | 147 | 147 | 227 | 227 | 301 | 301 |
| *Male 4* | 294 | 306 | 283 | 283 | 225 | 232 | 145 | 145 | 147 | 147 | 219 | 227 | 301 | 301 |
| juvenile | 294 | 306 | 283 | 283 | 225 | 232 | 145 | 145 | 147 | 147 | 219 | 227 | 301 | 301 |
| juvenile | 294 | 306 | 283 | 283 | 225 | 232 | 145 | 145 | 147 | 147 | 219 | 227 | 301 | 301 |
| juvenile | 294 | 306 | 283 | 283 | 225 | 232 | 145 | 145 | 147 | 147 | 227 | 227 | 301 | 301 |
| juvenile | 294 | 306 | 283 | 283 | 225 | 225 | 145 | 145 | 147 | 147 | 219 | 227 | 301 | 301 |
| juvenile | 294 | 306 | 283 | 283 | 225 | 232 | 145 | 145 | 147 | 147 | 227 | 227 | 301 | 301 |
| juvenile | 294 | 306 | 283 | 283 | 225 | 232 | 145 | 145 | 147 | 147 | 227 | 227 | 301 | 301 |
| juvenile | 294 | 306 | 283 | 283 | 225 | 225 | 145 | 145 | 147 | 147 | 219 | 227 | 301 | 301 |
| juvenile | 294 | 306 | 283 | 283 | 225 | 232 | 145 | 145 | 147 | 147 | 227 | 227 | 301 | 301 |
| juvenile | 294 | 306 | 283 | 283 | 225 | 232 | 145 | 145 | 147 | 147 | 219 | 227 | 301 | 301 |
| juvenile | 294 | 306 | 283 | 283 | 225 | 225 | 145 | 145 | 147 | 147 | 219 | 227 | 301 | 301 |
| *Female E* | 301 | 304 | 274 | 274 | 195 | 225 | 145 | 158 | 147 | 147 | 208 | 227 | 293 | 301 |
| *Male 5* | 294 | 301 | 278 | 281 | 225 | 229 | 145 | 158 | 147 | 161 | 208 | 208 | 293 | 295 |
| juvenile | 294 | 304 | 274 | 278 | 195 | 229 | 145 | 158 | 147 | 147 | 208 | 227 | 293 | 295 |
| juvenile | 294 | 304 | 274 | 278 | 195 | 225 | 145 | 158 | 147 | 147 | 208 | 227 | 295 | 301 |
| juvenile | 294 | 301 | 274 | 278 | 195 | 225 | 145 | 145 | 147 | 147 | 208 | 227 | 295 | 301 |
| juvenile | 301 | 301 | 274 | 281 | 195 | 225 | 145 | 158 | 147 | 161 | 208 | 227 | 295 | 301 |
| juvenile | 294 | 301 | 274 | 281 | 225 | 225 | 145 | 158 | 147 | 161 | 208 | 227 | 295 | 301 |
| juvenile | 301 | 301 | 274 | 281 | 225 | 229 | 145 | 145 | 147 | 161 | 208 | 208 | 293 | 295 |
| juvenile | 294 | 304 | 274 | 278 | 225 | 229 | 145 | 145 | 147 | 161 | 208 | 208 | 293 | 295 |
| juvenile | 294 | 304 | 274 | 278 | 225 | 229 | 145 | 158 | 147 | 161 | 208 | 208 | 293 | 295 |
| juvenile | 294 | 301 | 274 | 281 | 195 | 229 | 145 | 158 | 147 | 161 | 208 | 227 | 293 | 301 |
| juvenile | 294 | 301 | 274 | 281 | 195 | 229 | 145 | 158 | 147 | 161 | 208 | 227 | 293 | 301 |
